# Development and Internal Validation of a Side-Specific Nomogram Integrating mpMRI and Biopsy Features to Guide Nerve-Sparing Decision Making in Prostate Cancer with Capsular Contact

**DOI:** 10.3390/cancers18111788

**Published:** 2026-05-29

**Authors:** Yusuf Ahmed, Kateryna Diahovets, Tician Schnitzler, Lea Seiler, Alexander Cornelius, Fiona Burkhard, Georg Müller, Rainer Grobholz, Marco Cattaneo, Manuel Walter, Livio Nowak, Pirmin Wolfsgruber, Stephen Wyler, Lukas Prause, Maciej Kwiatkowski, Luca Afferi

**Affiliations:** 1 Department of Urology, Cantonal Hospital Aarau, 5001 Aarau, Switzerland; 2 Institute of Pathology, Cantonal Hospital Aarau, 5001 Aarau, Switzerland; 3 Department of Radiology, Cantonal Hospital Aarau, 5001 Aarau, Switzerland; 4 Medical Faculty, University of Zurich, 8032 Zurich, Switzerland; 5 Department of Clinical Research and Statistics, University of Basel, 4003 Basel, Switzerland; 6 Department of Urology, Hospital of Bozen (SABES-ASDAA), Teaching Hospital of Paracelsus Medical University (PMU), 39100 Bozen-Bolzano, Italy; 7 Department of Urology, Hospital Thun, 3600 Thun, Switzerland; livio.nowak@spitalstsag.ch; 8 Medical Faculty, University of Basel, 4003 Basel, Switzerland; 9 Department of Urology, Academic Hospital Braunschweig, 38126 Braunschweig, Germany

**Keywords:** capsular contact length, extracapsular extension, multiparametric magnetic resonance imaging, nerve-sparing surgery, nomogram, prostate cancer, radical prostatectomy, risk prediction

## Abstract

**Background**: Preoperative side-specific identification of extracapsular extension (ECE) is important for selecting an appropriate nerve-sparing strategy at radical prostatectomy. Patients with multiparametric magnetic resonance imaging (mpMRI)-defined capsular contact represent a clinically challenging subgroup because contact raises concern for ECE but does not uniformly indicate extraprostatic disease. We aimed to develop a side-specific nomogram for individualized ECE prediction and perform preliminary internal validation in this selected population. **Materials and Methods**: We retrospectively analyzed 323 prostate lobes from 286 patients with biopsy-proven, non-metastatic prostate cancer and mpMRI-defined capsular contact who underwent robot-assisted radical prostatectomy between 2015 and 2021 at a single institution. The dataset was randomly split into training (70%) and testing (30%) cohorts. Three multivariable logistic-regression models were developed and compared. Discrimination was assessed using the area under the receiver operating characteristic curve (AUC-ROC), calibration by intercept and slope, and clinical utility by decision curve analysis. A nomogram was derived from the best-performing model in the internal split-sample comparison. **Results**: Side-specific ECE was present in 110/323 lobes (34.1%). Among the candidate models, the forward-selection model showed the most favorable apparent performance, with an AUC-ROC of 0.85 in training and 0.83 in testing, together with good test-set calibration (intercept 0.24; slope 0.97). The final model included a capsular contact length ≥10 mm, percentage tumor involvement in positive biopsy cores, number of positive biopsy cores, and index lesion size. At a 10% predicted risk threshold, 32% of lobes were classified as low risk, with an observed ECE rate of about 5%. **Conclusions**: We developed a side-specific nomogram tailored to patients with mpMRI-defined capsular contact and performed preliminary internal validation. The model may aid preoperative side-specific risk assessment relevant to nerve-sparing planning, but external validation and assessment of clinical impact are required before clinical adoption.

## Simple Summary

Prostate cancer that appears to touch the outer edge of the prostate on magnetic resonance imaging presents a difficult surgical decision. Surgeons must balance complete cancer removal with preservation of the nerves that support urinary control and sexual function. However, not all patients with this imaging finding have cancer that has truly grown beyond the prostate. In this study, we developed a practical prediction tool that combines imaging findings with biopsy results to estimate, for each side of the prostate, the likelihood that the cancer has spread outside the gland. This tool may help surgeons plan treatment more precisely and support better discussions with patients before surgery. If validated in other cohorts, it could improve personalized surgical decision making and guide future research on prostate cancer care.

## 1. Introduction

Robot-assisted radical prostatectomy (RARP) remains a cornerstone curative treatment for clinically localized prostate cancer (PCa) [1]. Extracapsular extension (ECE), defined as tumor spread beyond the prostatic capsule into periprostatic tissue, is associated with higher rates of positive surgical margins and biochemical recurrence [2,3]. Conversely, wider surgical excision aimed at maximizing oncologic control may compromise urinary continence and erectile function. Accurate preoperative identification of side-specific ECE is therefore essential to balance oncologic safety with functional preservation [4].

Traditional prediction tools based on prostate-specific antigen (PSA), biopsy grade group, and clinical staging provide only moderate accuracy and limited side-specific guidance [5,6]. Multiparametric magnetic resonance imaging (mpMRI) has improved local staging [7,8,9], although subjective radiologic assessment of ECE remains variable and reader dependent [10,11]. Quantitative imaging features, particularly capsular contact length, have shown strong associations with ECE and have been incorporated into several MRI-based nomograms for side-specific prediction [12,13,14,15,16,17,18,19]. More recently, radiomics, artificial intelligence approaches, and updated MRI-integrated models have further refined preoperative risk assessment [20,21,22,23,24,25,26]. Nevertheless, clinical adoption remains limited by model complexity, inconsistent calibration across institutions, and the fact that most tools were developed in broader and more heterogeneous populations [16,17].

Recently, the European Society of Radiology (ESR) Essentials recommendations on MRI-based T-staging in prostate cancer proposed a more standardized framework, including use of the prefix “mr” for MRI-based staging and preference for the term “extraprostatic extension” over “extracapsular extension” [27]. In the present study, we retained the term ECE because it remains widely used in the literature on MRI-based nomograms and facilitates comparison with prior predictive studies [12,13,14,15,16,17,18,19,23,24,25,26].

Patients with mpMRI-defined capsular contact represent a distinct and clinically relevant subgroup, because capsular contact raises concern for ECE but does not uniformly indicate extraprostatic disease. In this setting, surgeons face substantial uncertainty when balancing oncologic control against functional preservation. Therefore, the aim of the present study was to develop a side-specific nomogram and perform preliminary internal validation using a split-sample approach to predict ECE and inform preoperative side-specific risk assessment relevant to nerve-sparing planning in this selected population.

## 2. Materials and Methods

We retrospectively analyzed 323 prostate lobes from 286 patients with biopsy-proven, non-metastatic prostate cancer and mpMRI-defined capsular contact who underwent robot-assisted radical prostatectomy at a single institution between April 2015 and January 2021. The analysis was restricted to prostate lobes in which capsular contact was identified on preoperative mpMRI. Three multivariable logistic regression models were developed and compared, and a nomogram was derived from the model showing the most favorable apparent performance in the internal split-sample comparison.

The unit of analysis was the prostate lobe. Imaging and biopsy variables were assigned side-specifically for the lobe under evaluation. Imaging variables used for modeling included Prostate Imaging Reporting and Data System (PI-RADS) category, capsular contact length, and index lesion size. Radiological suspicion of ECE, prostate capsule infiltration, and seminal vesicle invasion were recorded descriptively but were not included as candidate predictors because unequivocal radiological evidence of local extension would usually argue against nerve sparing on the affected side in routine clinical practice. Side-specific biopsy variables included the number of positive cores, percentage tumor involvement in positive cores, perineural invasion, and highest International Society of Urological Pathology (ISUP) grade group. Histopathologic ECE on the corresponding side of the prostatectomy specimen served as the reference outcome. In patients contributing both lobes, each side was matched to the corresponding side-specific imaging, biopsy, and pathological findings.

### 2.1. Clinical and Biopsy Variables

Collected clinical variables included age at surgery, PSA, prostate volume measured on mpMRI, and PSA density. Biopsy-derived parameters comprised the total number of biopsy cores, number of positive cores, percentage tumor involvement in positive cores, presence of perineural invasion, and highest side-specific ISUP grade group [28]. Biopsy practice reflected routine clinical care during the study period, consisting of systematic biopsy and MRI–ultrasound fusion-targeted biopsies of suspicious lesions (PI-RADS 3–5) in MRI. The radiological index lesion was defined as the dominant mpMRI lesion on the side under evaluation. Capsular contact length was therefore measured for the lesion considered most relevant for local staging on that side. Targeted biopsy findings were considered together with systematic biopsy results. For cases without unequivocal lesion-level targeted biopsy confirmation, the analysis relied on side-specific biopsy positivity and side-specific pathological ECE as a clinically relevant surgical reference.

### 2.2. mpMRI Acquisition and Interpretation

Multiparametric MRI examinations were performed between April 2015 and January 2021 according to institutional protocols aligned with European Society of Urogenital Radiology (ESUR) recommendations and PI-RADS v2.0 (used until 2020) and v2.1 (implemented from 2021 onward) [7,8]. Imaging interpretation was conducted by two dedicated genitourinary radiologists. Radiological assessment included PI-RADS category, index lesion size, capsular contact length, suspicion of ECE, prostate capsule infiltration, and seminal vesicle infiltration. Capsular contact length was measured as the maximal linear extent of tumor contact with the prostatic capsule on axial images. For categorical analysis, capsular contact was dichotomized at 10 mm, a cutoff repeatedly associated with increased risk of ECE in prior mpMRI-based staging studies [13,15].

### 2.3. Pathological Assessment

Whole-mount prostatectomy specimens were processed and reviewed by an experienced genitourinary pathologist. ECE was defined as tumor extension beyond the prostatic capsule into periprostatic tissue and served as the reference standard. Pathological grading, lymph node status, surgical margin status, and presence of perineural invasion were recorded. The principal pathological endpoint was side-specific ECE. Direct lesion-level co-registration between the MRI index lesion, biopsy target, and exact microscopic site of ECE was not available for all cases; therefore, the analysis used side-specific radiological, biopsy, and pathological matching.

### 2.4. Statistical Analysis and Model Development

Continuous variables were summarized as medians with interquartile ranges (IQRs) and categorical variables as frequencies and percentages. Radiological suspicion of ECE, prostate capsule infiltration, and seminal vesicle invasion detected on mpMRI were not included as candidate predictors because their presence would generally argue against nerve-sparing on the affected side in routine clinical practice. Accordingly, the nomogram is intended for patients with mpMRI-defined capsular contact in whom the appropriateness of nerve sparing remains uncertain; it should not be applied to override unequivocal radiological evidence of extraprostatic extension or seminal vesicle invasion.

The dataset was randomly divided into a training cohort (70%) and an independent test cohort (30%) using stratified sampling to preserve the distribution of ECE; a fixed random seed was used to ensure reproducibility. Model development, including variable selection, was performed in the training cohort, whereas model performance was evaluated in both cohorts. This split-sample approach was prespecified as preliminary internal validation. We acknowledge that a single split provides only a limited assessment of optimism and stability compared with resampling-based validation, especially in datasets with a moderate number of outcome events. Missing data were limited. Model fitting was performed using complete-case analysis for the variables included in each model. For the final nomogram-based cutoff analyses, 316 of 323 lobes (97.8%) had complete data; the remaining 7 lobes were excluded because of missing values in percentage tumor involvement in positive biopsy cores. Given the low proportion of missingness, no imputation was performed.

Two complementary modeling strategies were applied. Data-driven predictor selection was performed using multivariable logistic regression with predefined candidate variables: age, number of positive biopsy cores, percentage tumor involvement in positive biopsy cores, PSA density, side-specific PI-RADS score, capsular contact length on mpMRI, index lesion size, biopsy ISUP grade group, and perineural invasion. Variable selection used Akaike information criterion (AIC)-based stepwise procedures, including backward elimination from the full model and forward selection from the null model. In addition, a clinically prespecified multivariable logistic regression model incorporating capsular contact length, grouped biopsy ISUP grade, number of positive cores, percentage tumor involvement in positive biopsy cores, and PSA density was fitted in the training cohort. Predicted probabilities from each model were then applied to the test cohort. Continuous predictors were retained on their original scales whenever possible, whereas capsular contact length was dichotomized at 10 mm because this threshold is well supported in the literature and facilitates clinical use.

Model discrimination was assessed using the area under the curve of the receiver operating characteristic curve (AUC-ROC), with 95% confidence intervals calculated by the DeLong method. Predictor effects were reported as odds ratios (ORs) with corresponding 95% confidence intervals. Calibration was assessed using calibration-in-the-large (intercept), calibration slope, and Brier score in both datasets and graphically with calibration plots. Clinical utility of the forward selection model was evaluated using decision curve analysis across threshold probabilities ranging from 0 to 1, with primary clinical interpretation focused on thresholds between 5% and 25%.

A nomogram was constructed using the Regression Modeling Strategies (RMS) framework based on the model showing the most favorable apparent performance in the internal split-sample comparison. Predicted probabilities were also used to generate a cutoff summary table reporting the proportion of lobes classified below and above predefined probability thresholds, stratified by observed histologic ECE status. These thresholds were intended to illustrate model behavior across clinically plausible risk levels and should be interpreted as hypothesis-generating rather than as recommended clinical decision rules. Because the primary clinical target was side-specific nerve-sparing decision making, analyses were conducted at the lobe level. This approach preserves the intended clinical target, but it may modestly underestimate uncertainty because some patients contributed to two lobes. Accordingly, model coefficients should be interpreted primarily for prediction rather than etiologic inference, and patient-clustered robustness analyses or mixed-effects re-fitting should be considered in external validation datasets. All statistical analyses were performed using R version 4.5.2 (R Foundation for Statistical Computing, Vienna, Austria).

## 3. Results

Side-specific ECE was present in 110/323 lobes (34.1%). Among the evaluated candidate models, the forward selection model showed the most favorable apparent performance in this split-sample analysis, with an AUC-ROC of 0.85 in the training cohort and 0.83 in the testing cohort, together with good calibration in the testing cohort (intercept 0.24; slope 0.97). The final model included capsular contact length ≥10 mm, percentage tumor involvement in positive biopsy cores, number of positive biopsy cores, and index lesion size. Using a 10% predicted-risk threshold, 32% of lobes were classified as low risk, with an observed ECE rate of about 5% in this subgroup.

Baseline characteristics are summarized in Table 1. In total, 323 prostate lobes from 286 patients in whom capsular contact was observed on mpMRI were included in the analysis. The median age of patients was 67 (IQR: 62–71) years. Median PSA was 7 (IQR: 4.8–11) ng/mL, and median PSA density was 0.2 (IQR: 0.1–0.3) ng/mL/cm^3^. On mpMRI, PI-RADS 4 and 5 lesions accounted for 125 (38.7%) and 189 (58.5%) cases, respectively. Radiological signs of capsular infiltration were present in 228 (70.6%) prostate lobes. Capsular contact length was ≥10 mm in 187 (57.9%) prostate lobes, and the median index lesion size was 16 mm. Biopsy findings demonstrated a median of three (IQR 2–4) positive cores, with a median percentage tumor involvement of 56% in positive biopsy cores. Side-specific ISUP grade group ≥3 was present in 135 (41.8%) lobes. Perineural invasion was identified in 75 (23.2%) lobes.

### 3.1. Intraoperative and Pathological Characteristics

Intraoperative and pathological characteristics are described in Table 2. Lymphadenectomy was performed in 319 (98.8%) cases. Nerve-sparing RARP was achieved in 220 (68.2%) prostate lobes, including unilateral nerve sparing in 120 (37.2%) and bilateral nerve sparing in 100 (31%). On final histopathological examination, side-specific ECE was identified in 110 lobes (34.1%). Pathological lymph node involvement was observed in 25 (7.7%) cases. Positive surgical margins were present in 69 (21.4%) prostatectomy specimens. Final pathological grading showed ISUP grade group ≥3 disease in 142 (43.9%) cases, while perineural invasion was detected in 288 (89.2%). Positive surgical margin status was reported descriptively but was not selected as the primary endpoint for model development because margin status is influenced by tumor extent as well as intraoperative surgical plane, nerve-sparing extent, and surgeon-specific technical factors.

### 3.2. Model Performance and Internal Validation

**Figure 1:**
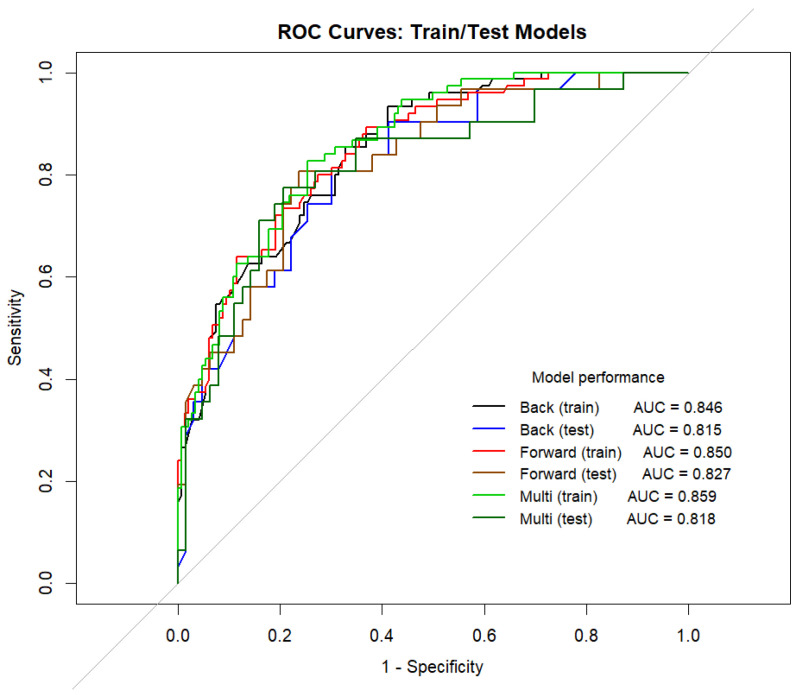
Comparison of the area under the curve of the receiver operating characteristic (AUC-ROC) curves describing sensitivity and specificity of the backward, forward and prespecified multivariable logistic regression analyses in the training (train) and testing (test) cohorts for the prediction of side-specific extracapsular extension in patients with prostate cancer and capsular contact at preoperative multiparametric magnetic resonance imaging (mpMRI).

The backward selection, forward selection, and prespecified multivariable models demonstrated good discrimination in both the training and testing cohorts (Figure 1). The forward selection model showed the smallest decrease in AUC-ROC between the training and testing cohorts (training AUC-ROC 0.85; testing AUC-ROC 0.83), indicating lower apparent optimism and greater stability on internal validation. Internal validation was based on a single random split-sample approach, which provides only a limited assessment of model optimism compared with resampling-based methods.

In the independent test dataset, the forward selection model also showed the most favorable calibration characteristics, with a calibration intercept of 0.24 and slope of 0.97, indicating good agreement between predicted and observed risks (Supplementary Table S1; calibration plots in Supplementary Figure S1). In contrast, the prespecified multivariable model exhibited a higher intercept (0.29) and lower calibration slope (0.82), suggesting greater overestimation of extreme predicted risks. Taken together, the forward selection model showed the most favorable apparent performance within this internal split-sample comparison and was therefore selected for nomogram construction.

Index lesion size was retained because its inclusion improved overall model fit according to AIC despite not reaching statistical significance for association with ECE (OR 1.04, 95% CI 0.99–1.08; *p* = 0.1). The predictor effects of the final forward-selection model are shown in Table 3.

### 3.3. Nomogram Development

**Figure 2:**
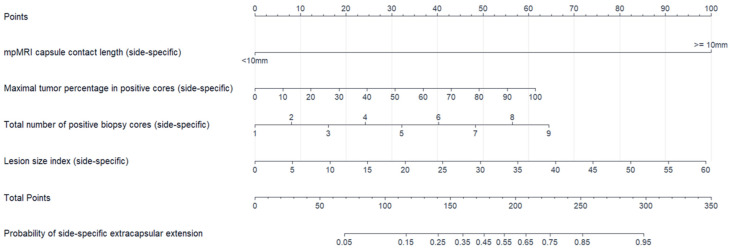
Nomogram predicting the side-specific probability of extracapsular extension (ECE) in patients with prostate cancer and capsular contact at multiparametric magnetic resonance imaging (mpMRI) using predictive variables identified with the forward selection model.

The final nomogram was based on the forward selection model and incorporated capsular contact length, percentage tumor involvement in positive biopsy cores, number of positive biopsy cores, and index lesion size to estimate the individualized side-specific probability of ECE (Figure 2). Capsular contact length contributed the greatest weight to the overall risk score.

How to use the nomogram: For the prostate side being assessed, identify the side-specific value for each predictor on the nomogram and read the corresponding points from the “Points” axis. Add all points to obtain the total score and locate it on the “Total Points” axis. Then, read the side-specific probability of extracapsular extension on the “Risk/Probability of ECE” axis.

### 3.4. Cutoff-Based Risk Stratification

Systematic evaluation of predicted probability thresholds is shown in Table 4. Seven prostate lobes had missing data for percentage tumor involvement in positive biopsy cores and were therefore excluded from the cutoff-based risk stratification analysis. Accordingly, Table 4 is based on 316 lobes with complete predictor data.

In this dataset, a 10% predicted probability threshold identified approximately 32% of prostate lobes as low risk, with an observed ECE rate of about 5% in this subgroup. This threshold may be useful as an illustrative reference point for preoperative risk stratification, but it should not be interpreted as a validated decision threshold for nerve-sparing surgery. Higher thresholds (15% and 20%) would classify a larger proportion of lobes as low risk, but at the cost of progressively higher observed rates of ECE (9% and 13%, respectively). Acceptable thresholds are expected to vary according to surgeon expertise, patient preferences, baseline functional status, and competing oncologic and functional priorities.

### 3.5. Clinical Utility

**Figure 3:**
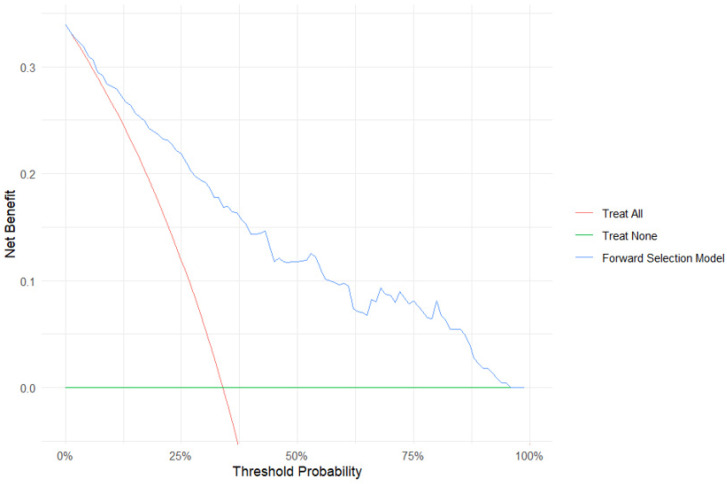
Decision curve analysis showing the net clinical benefit of the forward selection nomogram compared with treat-all and treat-none strategies across clinically relevant threshold probabilities of 5–25% for predicting side-specific extracapsular extension (ECE).

Clinical utility was evaluated using decision curve analysis (Figure 3), estimating net benefit of the forward selection model-derived nomogram in comparison with treat-all and treat-none strategies across threshold probabilities ranging from 0 to 1. Particular attention was given to threshold probabilities between 5% and 25%, reflecting clinically relevant ranges for nerve-sparing decision making. The nomogram provided higher net benefit than treat-all and treat-none strategies within this threshold range, suggesting potential value for identifying patients with lower side-specific risk of ECE. These findings reflect model-based risk stratification and do not, by themselves, establish a surgical decision rule. Rather, the nomogram is intended to complement radiological interpretation, surgeon judgment, and patient counseling.

## 4. Discussion

In this study, we developed a side-specific nomogram to predict ECE in patients with mpMRI-defined capsular contact undergoing RARP and performed preliminary internal validation using a split-sample approach. Despite radiologic capsular contact, histopathological ECE was identified in only one-third of prostate lobes, underscoring the potential to consider nerve sparing in selected patients. By integrating quantitative mpMRI findings with biopsy-derived tumor burden parameters, we derived a four-variable model with good discrimination and calibration in internal assessment. These performance estimates should be interpreted as preliminary rather than as definitive evidence of model transportability. The model is intended to complement, not replace, expert radiological assessment, surgeon judgment, and individualized patient counseling when planning side-specific nerve-sparing surgery.

Capsular contact length emerged as the strongest contributor to model performance. While capsular contact itself signals increased risk, its extent provides clinically relevant refinement. Within a population already enriched for suspected extracapsular disease, contact length functions primarily as a quantitative risk stratifier rather than a diagnostic marker. Dichotomization at 10 mm was chosen to facilitate clinical usability while preserving predictive performance.

Biopsy-derived tumor burden variables also improved prediction. The percentage of tumor involvement and the number of positive biopsy cores likely reflect local tumor volume and spatial distribution, complementing anatomical information derived from mpMRI. Together, these parameters capture the interaction between tumor biology and local morphology that underlies extracapsular spread. Because direct lesion-level proof that every MRI index lesion corresponded exactly to a positive targeted biopsy core and to the microscopic site of ECE was not available in all cases, our findings should be interpreted as side-specific rather than lesion-level prediction. Future studies restricted to biopsy-proven index lesions with lesion-level radiologic–pathologic co-registration may further refine model performance.

Biopsy ISUP grade was not retained in the final model. In this capsular-contact cohort, tumor burden and capsular interaction variables appeared to capture much of the prognostic information traditionally attributed to grade. Moreover, biopsy grading is susceptible to sampling variability and upgrading at prostatectomy, whereas quantitative imaging and burden metrics may more directly reflect the local processes underlying capsular invasion [12,13].

Clinically, the model identified a relevant low-risk subgroup despite suspicious imaging findings. Using a 10% predicted-risk threshold, approximately one-third of prostate lobes were classified as low risk, with a low observed rate of histological ECE (~5%) in this subgroup. This threshold may be useful as an illustrative reference point for preoperative stratification, but its appropriateness for guiding nerve-sparing surgery requires prospective evaluation because operative planning also depends on surgeon judgment, patient priorities, baseline sexual and urinary function, tumor location, and other clinical factors. Decision curve analysis suggested a potential net benefit across commonly considered threshold probabilities, indicating that individualized prediction may aid surgical risk stratification compared with uniform treatment strategies.

Our findings are broadly consistent with prior mpMRI-based nomograms combining imaging and biopsy variables for ECE prediction [15,16,17,18,19,23,24,25,26]. However, unlike most existing models, our nomogram was developed specifically in patients with mpMRI-defined capsular contact, a narrower subgroup in whom surgical decision making is particularly uncertain. Although radiomics and machine-learning approaches have shown promising predictive performance [20,21,22], simpler models based on standardized and routinely available variables may offer greater interpretability and easier clinical integration, provided that external validation confirms their performance. Importantly, the proposed tool should not be applied in patients with unequivocal radiological ECE or seminal vesicle invasion, in whom nerve sparing on the affected side would generally not be considered appropriate.

Several limitations warrant consideration. The retrospective single-center design may introduce selection bias and limits generalizability. Internal validation relied on a single 70/30 split-sample approach, which provides only a limited assessment of optimism and model stability compared with bootstrap or cross-validation methods. Therefore, the reported performance should be considered preliminary and require confirmation using resampling-based validation and independent external cohorts. In addition, the lobe-level analytical framework reflects the side-specific surgical decision the model is intended to support, but partial non-independence between left and right lobes may have resulted in optimistic uncertainty estimates and slightly inflated apparent model performance. The model should therefore be interpreted primarily as a clinical prediction tool rather than as evidence of causal predictor effects, and future validation studies should incorporate patient-clustered or mixed-effects sensitivity analyses. mpMRI interpretation was performed by experienced genitourinary radiologists, and inter-reader variability was not assessed. Changes in biopsy strategies over time, including increasing use of MRI-targeted biopsy [29], may influence tumor burden assessment and future calibration. Direct lesion-level radiologic–pathologic co-registration was not available for all cases, and the present model therefore predicts side-specific rather than lesion-specific ECE. Positive surgical margins were reported descriptively but were not modeled as a separate or combined endpoint because they are influenced by tumor extent and surgical technique. Functional and oncologic outcomes following model-guided nerve sparing, including surgical margin status and biochemical recurrence, were not evaluated and require prospective investigation.

In summary, this study provides a focused nomogram intended for side-specific risk estimation in patients with mpMRI-defined capsular contact in whom nerve-sparing remains clinically uncertain. The tool should be used, after external validation, as an adjunct to radiological assessment and surgical judgment rather than as an autonomous decision rule. External validation and prospective implementation studies are needed to determine whether model-guided nerve sparing improves functional outcomes while maintaining oncologic control.

## 5. Conclusions

We developed a nomogram specifically designed for patients with mpMRI-defined capsular contact and performed preliminary internal validation. Within this selected cohort, mpMRI-identified capsular contact was not uniformly associated with histologic ECE, and the proposed model may help refine preoperative side-specific risk assessment relevant to nerve-sparing planning during RARP. However, the nomogram should complement rather than replace radiological assessment, surgeon judgment, and individualized patient counseling. External validation is required before clinical implementation, and its effect on surgical decision making, functional outcomes, positive surgical margins, and oncologic control remains to be established.

## Figures and Tables

**Table 1:** Baseline characteristics of 323 prostate lobes harboring prostate cancer with capsular contact at mpMRI.

	Overall (*N* = 323)
**Age (years)**	67 (62–71)
**PSA [ng/mL]**	7 (4.8–11)
**PSA density [ng/mL/cm^3^]**	0.2 (0.1–0.3)
PI-RADS category *, *n* (%)	
PI-RADS 1	0
PI-RADS 2	2 (0.6)
PI-RADS 3	7 (2.2)
PI-RADS 4	125 (38.7)
PI-RADS 5	189 (58.5)
Prostate capsule infiltration at mpMRI *, *n* (%)	228 (70.6)
Prostate capsule contact length at mpMRI *, *n* (%)	
<10 mm	136 (42.1)
≥10 mm	187 (57.9)
Seminal vesicle infiltration at mpMRI *, *n* (%)	28 (8.7)
Index lesion size at mpMRI * [mm]	16 (12–21)
**Number of biopsies**	14 (13–14)
Number of positive biopsy cores *	3 (2–4)
Percentage of PCa in positive biopsy cores * [%]	56 (30–80)
**ISUP grade group in biopsy (side-specific), *n* (%)**	
ISUP 1	75 (23.2)
ISUP 2	112 (34.7)
ISUP 3	74 (22.9)
ISUP 4	31 (9.6)
ISUP 5	30 (9.3)
Perineural invasion in biopsy *, *n* (%)	75 (23.2)

Abbreviations: mpMRI = multiparametric magnetic resonance imaging; PSA = prostate-specific antigen; PI-RADS = Prostate Imaging Reporting and Data System; PCa = prostate cancer; ISUP = International Society of Urological Pathology; * side-specific.

**Table 2:** Intraoperative and pathological characteristics of prostate lobes with mpMRI-defined capsular contact treated by robot-assisted radical prostatectomy.

	Overall (*N* = 323)
**Lymphadenectomy, *n* (%)**	319 (98.8)
**Nerve sparing, *n* (%)**	
No	103 (31.9)
Unilateral	120 (37.2)
Bilateral	100 (31)
**Subtype of prostate cancer, *n* (%)**	
Prostatic adenocarcinoma	319 (98.8)
Ductal carcinoma of the prostate	4 (1.2)
**Extracapsular extension (side-specific), *n* (%)**	110 (34.1)
**Pathological N stage (pN1), *n* (%)**	25 (7.7)
**Number of LNs removed**	12 (8–17)
**Positive surgical margin, *n* (%)**	69 (21.4)
**ISUP, *n* (%)**	
ISUP 1	27 (8.4)
ISUP 2	154 (47.7)
ISUP 3	81 (25.1)
ISUP 4	15 (4.6)
ISUP 5	46 (14.2)
**Perineural invasion, *n* (%)**	288 (89.2)

Abbreviations: mpMRI = multiparametric magnetic resonance imaging*;* ISUP = International Society of Urological Pathology; LNs = lymph nodes.

**Table 3:** Multivariable logistic regression model for side-specific extracapsular extension.

	OR	95% CI	*p*-Value
**Prostate capsule contact length at mpMRI**			
<10 mm	Ref.	Ref.	Ref.
≥10 mm	8.48	3.74, 21.5	<0.001
Percentage of PCa in positive biopsy cores [%]	1.02	1.01, 1.03	0.003
**Absolute number of positive biopsy cores**	1.35	1.10, 1.68	0.005
Index lesion size [mm]	1.04	0.99, 1.08	0.12

Abbreviations: mpMRI = multiparametric magnetic resonance imaging; CI = confidence interval; OR = odds ratio; PCa = prostate cancer.

**Table 4:** Cutoff-based risk stratification for the forward selection model in lobes with complete predictor data (*N* = 316).

ECE Among Lobes Above Cutoff, *n* (%)	Lobes Above Cutoff, *n* (%)	ECE Among Lobes Below Cutoff, *n* (%)	Lobes Below Cutoff, *n* (%)	Predicted ECE-Risk Cutoff (%)
106 (100)	316 (100)	0 (0)	0 (0)	1
106 (100)	293 (93)	0 (0)	23 (7)	2
106 (100)	277 (88)	0 (0)	39 (12)	3
105 (99)	267 (84)	1 (1)	49 (16)	4
104 (98)	252 (80)	2 (2)	64 (20)	5
104 (98)	247 (78)	2 (2)	69 (22)	6
102 (96)	233 (74)	4 (4)	83 (26)	7
102 (96)	228 (72)	4 (4)	88 (28)	8
101 (95)	221 (70)	5 (5)	95 (30)	9
101 (95)	215 (68)	5 (5)	101 (32)	10
96 (91)	193 (61)	10 (9)	123 (39)	15
92 (87)	174 (55)	14 (13)	142 (45)	20

Abbreviations: ECE = extracapsular extension; *n* = number of lobes with complete predictor data.

## Data Availability

The data presented in this study are available from the corresponding author upon reasonable request. The data are not publicly available because of privacy and institutional restrictions.

## Supplementary Materials

The following supporting information can be downloaded at: https://www.mdpi.com/article/10.3390/cancers18111788/s1, Figure S1: Calibration plots showing the agreement between predicted and observed probabilities of side-specific extracapsular extension for each prediction model in training and testing datasets; Table S1: Calibration performance of prediction models: calibration intercept, slope, and Brier score compare the agreement between predicted and observed side-specific extracapsular extension risk across models in the training and testing cohorts.
