# Supplementary material for: Development and Internal Validation of a Side-Specific Nomogram Integrating mpMRI and Biopsy Features to Guide Nerve-Sparing Decision Making in Prostate Cancer with Capsular Contact
Source: Cancers (Basel). 2026 May 29;18(11):1788. doi: 10.3390/cancers18111788 (PMC13257312; doi:10.3390/cancers18111788)

Supplementary material

Supplementary Table S1. Calibration performance of prediction models: calibration intercept, slope, and Brier score compare the agreement between predicted and observed side-specific extracapsular extension risk across models in the training and testing cohorts.

Model	Dataset	Calibration Intercept	Calibration Slope	Brier Score
Backward	Train	-0.000	1.000	0.150
Backward	Test	0.209	0.940	0.161
Forward	Train	-0.000	1.000	0.147
Forward	Test	0.241	0.972	0.156
Multivariable	Train	-0.000	1.000	0.145
Multivariable	Test	0.295	0.826	0.159

Supplementary Figure S1. Calibration plots showing the agreement between predicted and observed probabilities of side-specific extracapsular extension for each prediction model in training and testing datasets.

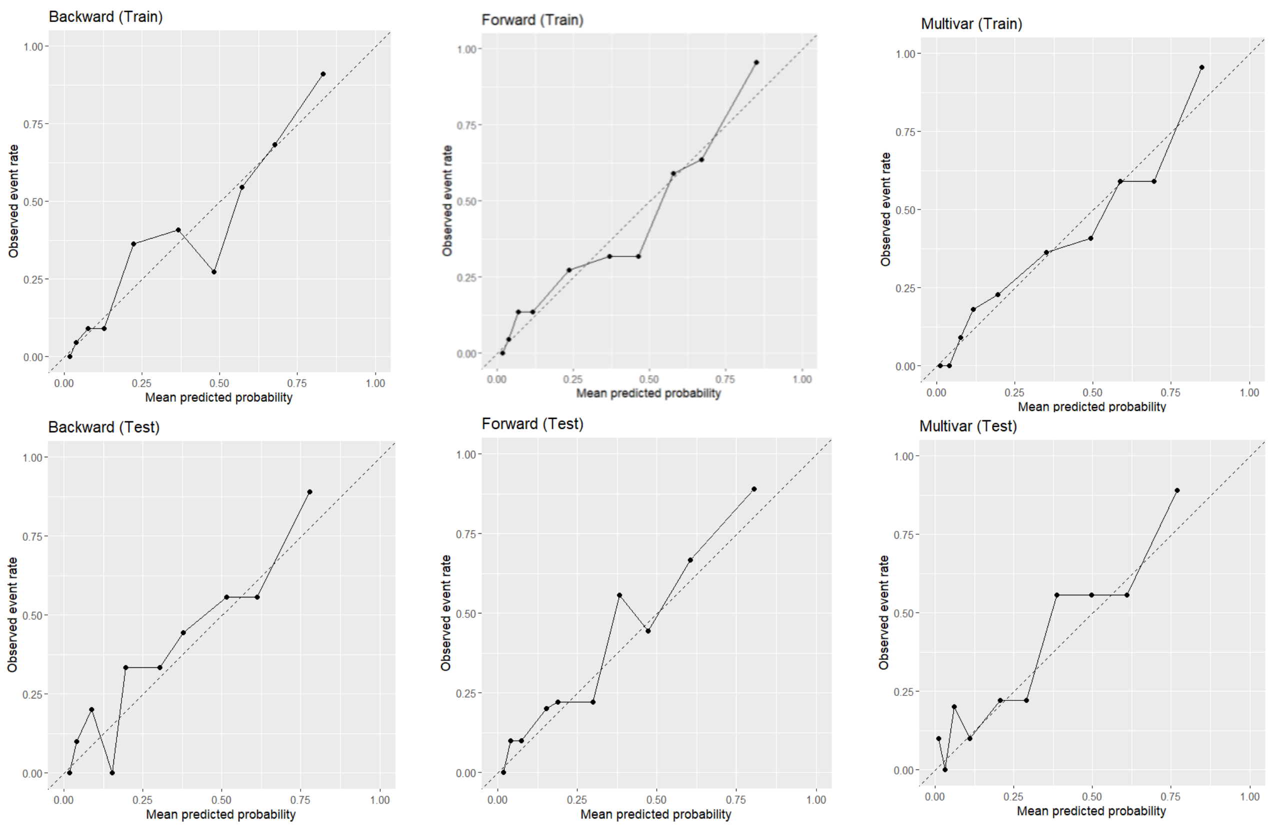

Supplement: Supplementary file 1 [file cancers-18-01788-s001.zip › cancers-4263950-supplementary.pdf]
